# Optimising the Conceptualisation of Context: Comment on "Stakeholder Perspectives of Attributes and Features of Context Relevant to Knowledge Translation in Health Settings: A Multi-country Analysis"

**DOI:** 10.34172/ijhpm.2022.6900

**Published:** 2022-09-26

**Authors:** Jo Rycroft-Malone, Lisa Rogers, Christopher R. Burton

**Affiliations:** ^1^Faculty of Health & Medicine, Lancaster University, Lancaster, UK; ^2^University College Dublin Centre for Interdisciplinary Research, Education, and Innovation in Health Systems (UCD IRIS), Health Sciences Centre, School of Nursing, Midwifery & Health Systems, University College Dublin, Dublin, Ireland; ^3^Health Sciences Centre, School of Nursing, Midwifery & Health Systems, University College Dublin, Dublin 4, Ireland.; ^4^School of Allied and Public Health Professions, Canterbury Christ Church University, Canterbury, UK.

**Keywords:** Context, Knowledge Translation, Implementation, De-implementation, Theory, Complexity

## Abstract

Context matters. Therefore, efforts to develop greater conceptual clarity are important for science and practice. In this commentary, we outline some key issues that were prompted by Squire’s et al.’s contribution. Specifically, we reinforce context as an interactive concept and therefore something that is hard to ‘pin down’, the problematic nature of conceptualising context in implementation and de-implementation, and a requirement for the development of culturally sensitive understandings. Finally, we suggest it is vital that continued investment into providing a more comprehensive list of determinants needs to be accompanied by an equal effort in developing practical methods and tools to support use and application.

## Introduction

 Context matters. It has been over two decades since scholars studying and writing about using evidence in healthcare practice noted the potentially important role that context plays.^1-3^ Over time the interest and scrutiny of the role of context in knowledge translation practice and research has resulted in an ever-growing empirical and theoretical evidence base. And yet, there are still many questions to answer. Squires et al^4^ reinforce the importance of context in knowledge translation activity but suggest that one of the reasons for a lack of progress in the field and the persistent challenge with improving outcomes for people, is that context has been poorly conceptualised. Squires et al^4^ argue that it is important to better understand the attributes of context to provide a more comprehensive framework to underpin an assessment toolbox and guide implementation efforts. As such, Squires et al^4^ offer a valuable conceptual contribution that has stimulated us to reflect on some key issues and questions about the role of context in knowledge translation research and practice.

## Conceptualising Context

 Squires et al^4^ rightly argue that to assess and report the influence of context, “this first requires conceptual clarity.” However, whilst conceptualising context is important, it is also challenging. The fundamental question is whether context is being conceived as a backdrop to action (separate from, but influencing), or an interacting and integral element in the knowledge translation process. We argue that reaching greater consensus on the factors that comprise context as Squires et al aspire to do, is challenging due to the dynamic nature of the construct.^5,6^ The authors allude to this concern when reporting the results, acknowledging that their participants “frequently discussed multiple attributes and features simultaneously” and “perceive[d] [context] in different ways.” Put another way, if we view context as an interacting element in the knowledge translation process, it becomes hard to pin down because it is not a static entity.

 Complexity science is a helpful lens with which to look at context. Rather than a static, predictable entity, context is characterised as an evolving, multi-faceted construct. This viewpoint is particularly applicable in health and care which is defined by its infinite combinations of activities, events, interactions, and outcomes.^7,8^ If we accept that context is not static and adopt a complexity science lens, which recognises context as a dynamic interacting construct, then arguably aiming for a standard representation of it might be flawed. Employing complexity theory when designing research would emphasise the interconnectedness of system components. This perspective will require researchers to recognise the interplay between contextual factors and account for the unexpected consequences resulting from the interacting elements. Adopting complexity theory may advance the field by progressing research away from simply identifying potential determinants, to research investigating the dynamic relationship between contextual features such as those listed by Squires and colleagues.^4^

 A dynamic and interacting view of context presents particular challenges to those who are in the practice of knowledge translation. However, methods are being advanced which may support change agents in the practice of knowledge translation by mapping the landscape of real-life contexts and the relationship between determinants.^9^ For example, using a constructivist approach and drawing on the Consolidated Framework for Implementation Research’s conceptualisation of context, the context coding framework facilitates an exploration of the relationships and dynamics between constructs over time.^9^ The context coding framework operates as a codebook enabling the collation of various data sources to capture context constructs at multiple levels of a system. The framework enables a blending of rapid evaluation and in-depth analysis. Rogers et al^9^ suggest that the coding framework can be used for documenting both the impact of context on the implementation effort, and the effect of the intervention on context longitudinally, before and during implementation processes. Employing such methods will likely assist with obtaining a deeper, more nuanced understanding of context, its influence, and interactions.

## The Role of Context in De-implementation

 A related domain of implementation research faces similar conceptual challenges: de-implementation or the de-adoption of low-value or ineffective healthcare practices. There may be an assumption that de-implementation processes mirror those of implementation or knowledge translation, albeit in reverse, with some transferability of implementation theory and frameworks. This was not an explicit focus of Squires and colleagues’ analysis, however it will be important to investigate the relevance of their conceptualisation of context for de-implementation research and practice. Whilst some aspects of context, such as financial considerations, may undoubtedly influence both implementation and de-implementation outcomes, there may be differences in how they influence those processes, including their relative importance. For example, our realist synthesis^10^ highlighted the significance of the emotional dimensions of de-implementation within contexts where there was a potential for strong patient-professional partnerships to develop over time. We suggest that in defining the determinants of context, we should also be considering how they might relate to both implementation and de-implementation.

## Context as an Internationally Relevant Concept

 The stakeholders in Squires and colleagues’ study were representative of four national contexts: Australia, Canada, the United Kingdom and the United States, each classified by the World Bank more correctly as a high-income country.^11^ Squires et al do make mention of the need for a similar study that tests the transferability of their findings to ‘developing countries.’ However, a more co-productive and inclusive approach to global research that investigates context may be required. For example, the assumption that similar participants, who in this study were mostly those working actively within implementation practice and research with a particular demographic profile, would be best able to contribute to similar research in low and middle-income countries needs further consideration. A more grounded and inclusive approach to research should be adopted that elaborates on, and accommodates differences across the political and policy contexts of health and care is needed. Characteristics to consider include social and cultural traditions around health and illness, the organisation and delivery of health and related services, and the histories and traditions of different health professional groups and allied health workers. In this way, a deeper understanding of relevant contextual influences, and importantly who can and should elaborate on them in implementation research and practice, will develop. It is also timely to critically reflect on the historical origins of many of the wider theories that inform knowledge translation, such as adult learning, organisational culture, and leadership, which are shaping our thinking on implementation context through a particular lens, which may not be culturally appropriate.

## Interaction Between Context and Intervention

 Building on the conceptualisation of knowledge translation as a process not an event, and context as an interacting element in that process, the implementation of a new intervention into routine practice is often challenged by a poor fit between intervention characteristics, the actions of implementers and the realities of everyday practice/context.^6^ Therefore, in addition to exploring the interrelatedness of contextual factors, we should also explore the interdependencies among these complex constructs. Recent research has begun to unravel the mechanisms underpinning these relationships. For example, our research exposed a bidirectional influence between context and implementation, revealing that these concepts dynamically interact, respond, and mutually evolve. In this study, consistent with the extant literature, implementation processes required adaptations to support the integration of change into routine practice.^12^ However, this influence was reciprocal. Determinants relating to implementation enhanced the surrounding context resulting in adaptions and improvements at a local level.^12^ Similarly, Haines et al^13^ emphasise the dynamic interplay between intervention implementation and effectiveness and the multi-level contexts in which they are implemented. These authors recommend the need for methodological advancements to better account for and attend to these complex interactions.^13^ As acknowledged by Squires and colleagues^4^ health system stakeholders have “tacit, first-hand knowledge of KT and the effects of context” on implementation. Therefore, harnessing this experience and incorporating user-centred designs when planning interventions, preparing contexts, and informing implementation strategies will likely support the successful translation of evidence into routine practice.

## Practical Application

 While the theoretical evidence base for conceptualising context has grown,^1,2,14^ there has been less attention to what is required to operationalise the concept for practical use. Whilst some conceptualisations of context have been translated into assessment tools (eg, Alberta Context Tool, Context Assessment Index) the practical application of such methods (ie, their usability and usefulness) among frontline stakeholders remains unclear. Innovative methods have been developed (eg, the Consolidated Framework for Implementation Research game^15^) to support the accessibility of implementation research in practice. However, future researchers may benefit from leveraging more active and earlier stakeholder engagement, and rather than use a developed tool *on* staff, co-produce a method to assess context *with* stakeholders. By working *with* knowledge users including change agents, the accessibility of the content will likely improve and may ultimately bridge the gap between our theoretical understanding context (ie, academic implementation science) and the practical application of this knowledge in practice (ie, real-world implementation planning and practice).

## Conclusion

 Refining our understanding of the concept of context is important for scientific and practical reasons. We have highlighted that the dynamic and therefore malleable nature of context poses both challenges and opportunities. It is critical that a more comprehensive understanding is inclusive and culturally sensitive, and that it is driven by an appreciation that given the interacting nature of context, at best, a measure of context will only ever provide a snapshot in time. Finally, we argue it is vital that continued investment into providing conceptual clarity and a more comprehensive list of determinants needs to be accompanied by an equal effort in developing practical methods and tools to support use and application.

## Ethical issues

 Not applicable.

## Competing interests

 Authors declare that they have no competing interests.

## Authors’ contributions

 All authors contributed to the concept, drafting and finalising of the manuscript.
